# Tris[2-(1*H*-imidazol-2-yl)imidazol-1-ido]cobalt(III)

**DOI:** 10.1107/S1600536810011785

**Published:** 2010-04-10

**Authors:** Qi Ma, Miaoli Zhu, Sisi Feng, Liping Lu

**Affiliations:** a Institute of Molecular Science, Key Laboratory of Chemical Biology and Molecular Engineering of the Education Ministry, Shanxi University, Taiyuan, Shanxi 030006, People’s Republic of China; b College of Chemistry and Chemical Engineering, Shanxi Datong University, Datong, Shanxi 037009, People’s Republic of China

## Abstract

In the title compound, [Co(C_6_H_5_N_4_)_3_], the Co^III^ atom adopts a distorted octa­hedral CoN_6_ coordination geometry, arising from three *N*,*N*′-bidentate deprotonated 2,2′-biimidazole ligands. The dihedral angles between the five-membered rings of the ligands are 4.1 (2), 9.4 (2) and 10.5 (2)°. In the crystal, mol­ecules are linked by N—H⋯N hydrogen bonds, generating a layered network lying in (11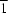).

## Related literature

For related structures, see: Tadokoro & Nakasuji (2000 ▶); Ye *et al.* (2005 ▶); Zhang *et al.* (2008 ▶). 
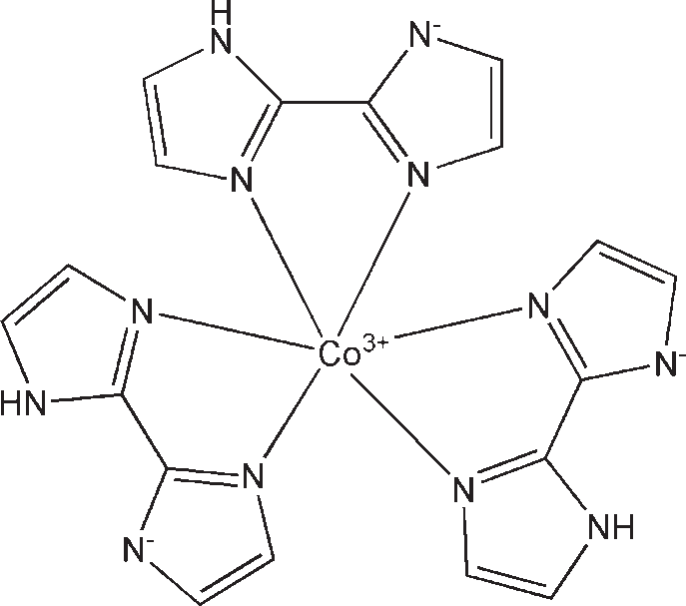


## Experimental

### 

#### Crystal data

[Co(C_6_H_5_N_4_)_3_]
*M* *_r_* = 458.35
Monoclinic, 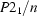

*a* = 12.299 (3) Å
*b* = 12.524 (3) Å
*c* = 12.932 (3) Å
β = 97.773 (4)°
*V* = 1973.6 (8) Å^3^
*Z* = 4
Mo *K*α radiation
μ = 0.90 mm^−1^
*T* = 293 K
0.5 × 0.4 × 0.3 mm

#### Data collection

Bruker SMART CCD diffractometer
Absorption correction: multi-scan (*SADABS*; Bruker, 2000 ▶) *T*_min_ = 0.654, *T*_max_ = 0.762
10212 measured reflections
3728 independent reflections
2358 reflections with *I* > 2σ(*I*)
*R*_int_ = 0.046

#### Refinement

*R*[*F* ^2^ > 2σ(*F* ^2^)] = 0.041
*wR*(*F* ^2^) = 0.138
*S* = 0.98
3728 reflections
280 parameters
H-atom parameters constrained
Δρ_max_ = 0.48 e Å^−3^
Δρ_min_ = −0.49 e Å^−3^

### 

Data collection: *SMART* (Bruker, 2000 ▶); cell refinement: *SAINT* (Bruker, 2000 ▶); data reduction: *SAINT*; program(s) used to solve structure: *SHELXS97* (Sheldrick, 2008 ▶); program(s) used to refine structure: *SHELXL97* (Sheldrick, 2008 ▶); molecular graphics: *SHELXTL* (Sheldrick, 2008 ▶); software used to prepare material for publication: *SHELXTL*.

## Supplementary Material

Crystal structure: contains datablocks I, global. DOI: 10.1107/S1600536810011785/hb5377sup1.cif

Structure factors: contains datablocks I. DOI: 10.1107/S1600536810011785/hb5377Isup2.hkl

Additional supplementary materials:  crystallographic information; 3D view; checkCIF report

## Figures and Tables

**Table:** 

Co1—N4	1.917 (3)
Co1—N3	1.922 (3)
Co1—N6	1.926 (3)
Co1—N1	1.929 (3)
Co1—N5	1.941 (3)
Co1—N2	1.944 (3)

**Table:** 

N4—Co1—N3	82.54 (12)
N6—Co1—N5	81.67 (13)
N1—Co1—N2	82.18 (12)

**Table 2:** Hydrogen-bond geometry (Å, °)

*D*—H⋯*A*	*D*—H	H⋯*A*	*D*⋯*A*	*D*—H⋯*A*
N11—H6⋯N12^i^	0.86	1.95	2.808 (4)	172
N7—H11⋯N8^ii^	0.86	1.99	2.814 (4)	159
N9—H19⋯N10^iii^	0.86	1.95	2.796 (4)	169

Symmetry codes: (i) 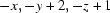; (ii) 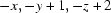; (iii) 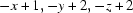.

**Fig. 1.**
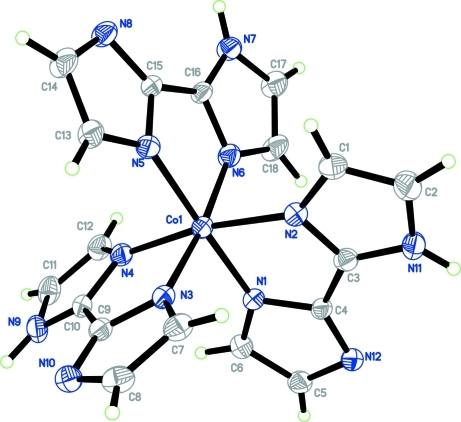
A view of the structure of (I) with displacement ellipsoids drawn at the 30% probability level.

**Fig. 2.**
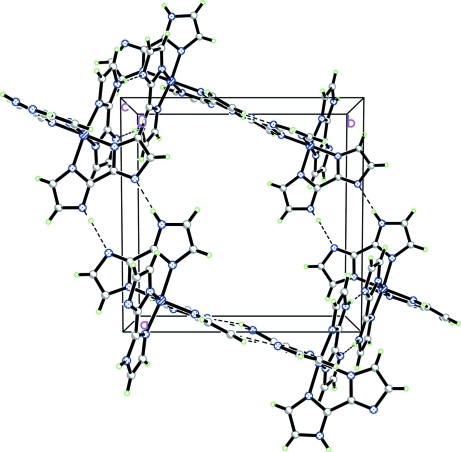
hydrogen bond interaction in neighboring molecules.

## supplementary crystallographic information

### Comment

The neutral molecule 2,2'-biimidazole (H_2_biim) and its monoanionic derivative(Hbiim^-^) is a particular organic target for construction of hybrid materials. Its molecular moieties possess a double property. Namely they can be coordinated to metal centres and can act as a donor in hydrogen bonding interactions (Tadokoro & Nakasuji, 2000). The crystal structure of (I) is reported here.

The X-ray crystallographic analysis shows that the molecule of the compound (I) consists of three Hbiim^-^ and one Co^3+^ (Fig. 1).  The Co^3+^ ion adopted octahedron coordination geometry, and coordinated with three Hbiim^-^ anion. Average bond distance Co—N is 1.93 (3) Å, shorter than Co—N bond distance found in related structures, i.e. 2.116 (2)-2.118Å in [Co(H_2_biim)_2_(1,2-bdc)] (Ye *et al.*, 2005), 2.1563 (18)Å , in diaquabis(2,2'-biimidazole)cobalt(II) dichloride (Zhang *et al.*, 2008). In the crystalline state, the neighboring molecules are linked furtherly by N—H···N hydrogen bonding forming supermolecular structure(Fig. 2).

### Experimental

CoCl_2_.6H_2_O (0.1904 g, 0.8 mmol), biimidazole (0.107 g, 1 mmol), and water (10 ml) were added to an aqueous solution (5 ml) containing NaN_3_ (0.028 g,0.4 mmol). The resulting mixture was further stirred for 15 min in air, and then transferred and sealed in a 20 ml Teflon-lined reactor, which was heated at 423 K for 4 days and then cooled to room temperature at a rate of 5 K h^-1^. Red blocks of (I) were obtained and washed with water.

### Refinement

H atoms attached to C and N atoms of (I) were placed in geometrically idealized positions (C—H = 0.93Å, N—H = 0.86Å and constrained to ride on their parent atoms.

### Crystal data

**Table:**

[Co(C_6_H_5_N_4_)_3_]	*F*(000) = 936
*M**_r_* = 458.35	*D*_x_ = 1.543 Mg m^−^^3^
Monoclinic, *P*2_1_/*n*	Mo *K*α radiation, λ = 0.71073 Å
Hall symbol: -P 2yn	Cell parameters from 10212 reflections
*a* = 12.299 (3) Å	θ = 2.3–20.5°
*b* = 12.524 (3) Å	µ = 0.90 mm^−^^1^
*c* = 12.932 (3) Å	*T* = 293 K
β = 97.773 (4)°	Block, red
*V* = 1973.6 (8) Å^3^	0.5 × 0.4 × 0.3 mm
*Z* = 4	

### Data collection

**Table:**

Bruker SMART CCD diffractometer	3728 independent reflections
Radiation source: fine-focus sealed tube	2358 reflections with *I* > 2σ(*I*)
graphite	*R*_int_ = 0.046
ω scans	θ_max_ = 25.7°, θ_min_ = 2.1°
Absorption correction: multi-scan (*SADABS*; Bruker, 2000)	*h* = −14→15
*T*_min_ = 0.654, *T*_max_ = 0.762	*k* = −15→13
10212 measured reflections	*l* = −15→13

### Refinement

**Table:**

Refinement on *F*^2^	Primary atom site location: structure-invariant direct methods
Least-squares matrix: full	Secondary atom site location: difference Fourier map
*R*[*F*^2^ > 2σ(*F*^2^)] = 0.041	Hydrogen site location: inferred from neighbouring sites
*wR*(*F*^2^) = 0.138	H-atom parameters constrained
*S* = 0.98	*w* = 1/[σ^2^(*F*_o_^2^) + (0.0798*P*)^2^] where *P* = (*F*_o_^2^ + 2*F*_c_^2^)/3
3728 reflections	(Δ/σ)_max_ < 0.001
280 parameters	Δρ_max_ = 0.48 e Å^−^^3^
0 restraints	Δρ_min_ = −0.49 e Å^−^^3^

### Special details

**Table:**

Geometry. All esds (except the esd in the dihedral angle between two l.s. planes) are estimated using the full covariance matrix. The cell esds are taken into account individually in the estimation of esds in distances, angles and torsion angles; correlations between esds in cell parameters are only used when they are defined by crystal symmetry. An approximate (isotropic) treatment of cell esds is used for estimating esds involving l.s. planes.
Refinement. Refinement of *F*^2^ against ALL reflections. The weighted *R*-factor wR and goodness of fit *S* are based on *F*^2^, conventional *R*-factors *R* are based on *F*, with *F* set to zero for negative *F*^2^. The threshold expression of *F*^2^ > σ(*F*^2^) is used only for calculating *R*-factors(gt) etc. and is not relevant to the choice of reflections for refinement. *R*-factors based on *F*^2^ are statistically about twice as large as those based on *F*, and *R*- factors based on ALL data will be even larger.

### Fractional atomic coordinates and isotropic or equivalent isotropic displacement parameters (Å^2^)

**Table:**

	*x*	*y*	*z*	*U*_iso_*/*U*_eq_	
Co1	0.14643 (4)	0.83171 (4)	0.84581 (4)	0.03246 (19)	
N1	0.1905 (2)	0.8669 (2)	0.7123 (2)	0.0325 (7)	
N2	0.0080 (2)	0.8920 (2)	0.7814 (2)	0.0348 (7)	
N3	0.2012 (2)	0.9650 (2)	0.9055 (2)	0.0324 (7)	
N4	0.2936 (2)	0.7858 (2)	0.8922 (2)	0.0344 (7)	
N5	0.1019 (2)	0.7861 (2)	0.9773 (2)	0.0348 (8)	
N6	0.0917 (2)	0.6922 (2)	0.8032 (3)	0.0365 (8)	
C5	0.2563 (3)	0.9108 (3)	0.5682 (3)	0.0400 (10)	
H2	0.3051	0.9191	0.5197	0.048*	
C6	0.2817 (3)	0.8656 (3)	0.6656 (3)	0.0396 (10)	
H1	0.3498	0.8388	0.6939	0.048*	
N12	0.1482 (2)	0.9419 (2)	0.5534 (2)	0.0360 (8)	
C4	0.1135 (3)	0.9131 (3)	0.6429 (3)	0.0308 (8)	
C3	0.0105 (3)	0.9241 (3)	0.6834 (3)	0.0343 (9)	
N11	−0.0888 (2)	0.9582 (2)	0.6405 (3)	0.0433 (9)	
H6	−0.1059	0.9829	0.5783	0.052*	
C2	−0.1578 (3)	0.9462 (4)	0.7141 (4)	0.0516 (12)	
H7	−0.2322	0.9627	0.7060	0.062*	
C1	−0.0984 (3)	0.9061 (3)	0.8007 (3)	0.0435 (10)	
H8	−0.1249	0.8905	0.8630	0.052*	
C18	0.0637 (4)	0.6367 (3)	0.7131 (3)	0.0477 (11)	
H9	0.0782	0.6578	0.6473	0.057*	
C17	0.0115 (4)	0.5462 (3)	0.7345 (3)	0.0493 (11)	
H10	−0.0153	0.4939	0.6866	0.059*	
N7	0.0051 (3)	0.5451 (2)	0.8392 (3)	0.0397 (8)	
H11	−0.0243	0.4963	0.8731	0.048*	
C16	0.0534 (3)	0.6349 (3)	0.8788 (3)	0.0322 (9)	
C15	0.0665 (3)	0.6837 (3)	0.9789 (3)	0.0306 (8)	
N8	0.0546 (3)	0.6485 (2)	1.0734 (2)	0.0412 (8)	
C14	0.0858 (3)	0.7350 (3)	1.1374 (3)	0.0477 (11)	
H15	0.0868	0.7361	1.2094	0.057*	
C13	0.1150 (3)	0.8185 (3)	1.0787 (3)	0.0392 (10)	
H16	0.1393	0.8852	1.1036	0.047*	
C12	0.3600 (3)	0.7001 (3)	0.8784 (3)	0.0429 (10)	
H17	0.3368	0.6345	0.8496	0.051*	
C11	0.4643 (3)	0.7274 (3)	0.9139 (3)	0.0469 (11)	
H18	0.5256	0.6836	0.9151	0.056*	
N9	0.4642 (3)	0.8312 (2)	0.9480 (3)	0.0413 (8)	
H19	0.5204	0.8683	0.9734	0.050*	
C10	0.3598 (3)	0.8634 (3)	0.9339 (3)	0.0331 (9)	
C9	0.3067 (3)	0.9641 (3)	0.9483 (3)	0.0333 (9)	
N10	0.3413 (2)	1.0555 (2)	0.9935 (3)	0.0368 (8)	
C8	0.2507 (3)	1.1201 (3)	0.9770 (3)	0.0419 (10)	
H23	0.2482	1.1907	0.9988	0.050*	
C7	0.1643 (3)	1.0652 (3)	0.9236 (3)	0.0394 (9)	
H24	0.0939	1.0913	0.9034	0.047*	

### Atomic displacement parameters (Å^2^)

**Table:**

	*U*^11^	*U*^22^	*U*^33^	*U*^12^	*U*^13^	*U*^23^
Co1	0.0365 (3)	0.0314 (3)	0.0284 (3)	−0.0033 (2)	0.0010 (2)	0.0038 (2)
N1	0.0327 (16)	0.0320 (16)	0.0321 (19)	−0.0013 (13)	0.0023 (14)	0.0052 (14)
N2	0.0354 (17)	0.0352 (18)	0.033 (2)	−0.0026 (14)	0.0028 (14)	0.0041 (15)
N3	0.0359 (17)	0.0304 (17)	0.030 (2)	−0.0012 (13)	0.0013 (14)	0.0040 (13)
N4	0.0396 (17)	0.0262 (16)	0.036 (2)	−0.0012 (14)	−0.0009 (15)	0.0041 (14)
N5	0.0377 (17)	0.0316 (17)	0.035 (2)	−0.0041 (14)	0.0021 (15)	0.0008 (14)
N6	0.0437 (18)	0.0348 (17)	0.030 (2)	−0.0040 (14)	0.0017 (15)	0.0001 (14)
C5	0.040 (2)	0.046 (2)	0.035 (3)	−0.0064 (18)	0.0082 (19)	0.0010 (19)
C6	0.034 (2)	0.042 (2)	0.041 (3)	−0.0004 (17)	0.0028 (19)	0.0017 (19)
N12	0.0379 (18)	0.0370 (18)	0.032 (2)	−0.0054 (14)	0.0008 (15)	0.0026 (14)
C4	0.0361 (19)	0.0306 (19)	0.025 (2)	−0.0051 (16)	0.0013 (16)	0.0005 (16)
C3	0.038 (2)	0.033 (2)	0.031 (2)	−0.0034 (17)	0.0056 (18)	0.0003 (17)
N11	0.0374 (18)	0.055 (2)	0.036 (2)	0.0019 (15)	0.0008 (16)	0.0097 (16)
C2	0.034 (2)	0.071 (3)	0.051 (3)	0.004 (2)	0.009 (2)	0.010 (2)
C1	0.039 (2)	0.057 (3)	0.037 (3)	−0.0025 (19)	0.0129 (19)	0.006 (2)
C18	0.074 (3)	0.046 (2)	0.024 (2)	−0.006 (2)	0.010 (2)	−0.0032 (19)
C17	0.072 (3)	0.046 (3)	0.029 (3)	−0.013 (2)	0.003 (2)	−0.0085 (19)
N7	0.051 (2)	0.0336 (18)	0.034 (2)	−0.0088 (15)	0.0043 (16)	−0.0024 (15)
C16	0.040 (2)	0.033 (2)	0.024 (2)	−0.0040 (16)	0.0040 (17)	0.0020 (16)
C15	0.0329 (19)	0.030 (2)	0.028 (2)	−0.0031 (15)	0.0023 (16)	0.0038 (16)
N8	0.056 (2)	0.0400 (19)	0.027 (2)	−0.0081 (15)	0.0056 (17)	0.0011 (15)
C14	0.069 (3)	0.051 (3)	0.024 (2)	−0.007 (2)	0.010 (2)	−0.004 (2)
C13	0.051 (2)	0.037 (2)	0.029 (2)	−0.0044 (18)	0.0035 (19)	−0.0078 (18)
C12	0.056 (3)	0.030 (2)	0.041 (3)	0.0033 (18)	0.000 (2)	0.0027 (18)
C11	0.050 (2)	0.042 (3)	0.046 (3)	0.014 (2)	−0.003 (2)	0.006 (2)
N9	0.0407 (18)	0.0404 (19)	0.039 (2)	0.0016 (15)	−0.0083 (16)	−0.0009 (15)
C10	0.033 (2)	0.034 (2)	0.030 (2)	−0.0009 (16)	−0.0031 (17)	0.0073 (17)
C9	0.038 (2)	0.031 (2)	0.030 (2)	−0.0019 (16)	0.0032 (18)	0.0034 (16)
N10	0.0440 (18)	0.0334 (18)	0.034 (2)	−0.0023 (14)	0.0071 (15)	−0.0058 (14)
C8	0.053 (2)	0.033 (2)	0.043 (3)	−0.0008 (19)	0.016 (2)	−0.0025 (19)
C7	0.043 (2)	0.036 (2)	0.041 (3)	0.0060 (18)	0.0120 (19)	0.0044 (18)

### Geometric parameters (Å, °)

**Table:**

Co1—N4	1.917 (3)	C2—C1	1.348 (5)
Co1—N3	1.922 (3)	C2—H7	0.9300
Co1—N6	1.926 (3)	C1—H8	0.9300
Co1—N1	1.929 (3)	C18—C17	1.350 (5)
Co1—N5	1.941 (3)	C18—H9	0.9300
Co1—N2	1.944 (3)	C17—N7	1.367 (5)
N1—C6	1.344 (4)	C17—H10	0.9300
N1—C4	1.345 (4)	N7—C16	1.341 (4)
N2—C3	1.333 (5)	N7—H11	0.8600
N2—C1	1.376 (4)	C16—C15	1.420 (5)
N3—C9	1.340 (4)	C15—N8	1.326 (5)
N3—C7	1.365 (4)	N8—C14	1.386 (5)
N4—C10	1.335 (5)	C14—C13	1.369 (5)
N4—C12	1.374 (4)	C14—H15	0.9300
N5—C15	1.356 (4)	C13—H16	0.9300
N5—C13	1.361 (5)	C12—C11	1.347 (5)
N6—C16	1.349 (5)	C12—H17	0.9300
N6—C18	1.361 (5)	C11—N9	1.373 (5)
C5—N12	1.373 (4)	C11—H18	0.9300
C5—C6	1.378 (5)	N9—C10	1.334 (5)
C5—H2	0.9300	N9—H19	0.8600
C6—H1	0.9300	C10—C9	1.443 (5)
N12—C4	1.336 (5)	C9—N10	1.329 (4)
C4—C3	1.442 (5)	N10—C8	1.370 (5)
C3—N11	1.341 (5)	C8—C7	1.372 (5)
N11—C2	1.367 (5)	C8—H23	0.9300
N11—H6	0.8600	C7—H24	0.9300
			
N4—Co1—N3	82.54 (12)	C1—C2—H7	126.2
N4—Co1—N6	95.52 (13)	N11—C2—H7	126.2
N3—Co1—N6	173.01 (13)	C2—C1—N2	108.6 (4)
N4—Co1—N1	88.88 (12)	C2—C1—H8	125.7
N3—Co1—N1	92.05 (13)	N2—C1—H8	125.7
N6—Co1—N1	94.62 (13)	C17—C18—N6	109.0 (4)
N4—Co1—N5	90.23 (13)	C17—C18—H9	125.5
N3—Co1—N5	91.61 (13)	N6—C18—H9	125.5
N6—Co1—N5	81.67 (13)	C18—C17—N7	107.7 (4)
N1—Co1—N5	176.08 (12)	C18—C17—H10	126.2
N4—Co1—N2	170.39 (13)	N7—C17—H10	126.2
N3—Co1—N2	94.22 (12)	C16—N7—C17	106.8 (3)
N6—Co1—N2	88.73 (13)	C16—N7—H11	126.6
N1—Co1—N2	82.18 (12)	C17—N7—H11	126.6
N5—Co1—N2	98.93 (12)	N7—C16—N6	110.4 (3)
C6—N1—C4	105.1 (3)	N7—C16—C15	134.4 (3)
C6—N1—Co1	138.8 (3)	N6—C16—C15	115.1 (3)
C4—N1—Co1	116.0 (2)	N8—C15—N5	113.9 (3)
C3—N2—C1	106.1 (3)	N8—C15—C16	133.1 (3)
C3—N2—Co1	113.1 (2)	N5—C15—C16	113.0 (3)
C1—N2—Co1	140.5 (3)	C15—N8—C14	103.5 (3)
C9—N3—C7	105.2 (3)	C13—C14—N8	109.8 (4)
C9—N3—Co1	115.2 (2)	C13—C14—H15	125.1
C7—N3—Co1	139.5 (3)	N8—C14—H15	125.1
C10—N4—C12	106.3 (3)	N5—C13—C14	107.5 (3)
C10—N4—Co1	114.0 (2)	N5—C13—H16	126.2
C12—N4—Co1	138.2 (3)	C14—C13—H16	126.2
C15—N5—C13	105.3 (3)	C11—C12—N4	108.2 (3)
C15—N5—Co1	114.9 (3)	C11—C12—H17	125.9
C13—N5—Co1	138.2 (3)	N4—C12—H17	125.9
C16—N6—C18	106.1 (3)	C12—C11—N9	107.9 (3)
C16—N6—Co1	114.7 (3)	C12—C11—H18	126.1
C18—N6—Co1	138.3 (3)	N9—C11—H18	126.1
N12—C5—C6	109.9 (3)	C10—N9—C11	106.6 (3)
N12—C5—H2	125.1	C10—N9—H19	126.7
C6—C5—H2	125.1	C11—N9—H19	126.7
N1—C6—C5	107.8 (3)	N9—C10—N4	111.0 (3)
N1—C6—H1	126.1	N9—C10—C9	133.7 (3)
C5—C6—H1	126.1	N4—C10—C9	115.2 (3)
C4—N12—C5	102.7 (3)	N10—C9—N3	114.1 (3)
N12—C4—N1	114.6 (3)	N10—C9—C10	133.3 (3)
N12—C4—C3	133.6 (3)	N3—C9—C10	112.6 (3)
N1—C4—C3	111.8 (3)	C9—N10—C8	103.6 (3)
N2—C3—N11	110.7 (3)	N10—C8—C7	109.9 (3)
N2—C3—C4	116.8 (3)	N10—C8—H23	125.0
N11—C3—C4	132.5 (4)	C7—C8—H23	125.0
C3—N11—C2	107.0 (3)	N3—C7—C8	107.1 (3)
C3—N11—H6	126.5	N3—C7—H24	126.4
C2—N11—H6	126.5	C8—C7—H24	126.4
C1—C2—N11	107.6 (3)		
			
N4—Co1—N1—C6	−1.6 (4)	C1—N2—C3—C4	−177.6 (3)
N3—Co1—N1—C6	80.9 (4)	Co1—N2—C3—C4	−2.2 (4)
N6—Co1—N1—C6	−97.0 (4)	N12—C4—C3—N2	−175.1 (4)
N2—Co1—N1—C6	174.9 (4)	N1—C4—C3—N2	2.5 (5)
N4—Co1—N1—C4	−176.0 (3)	N12—C4—C3—N11	7.4 (7)
N3—Co1—N1—C4	−93.5 (3)	N1—C4—C3—N11	−174.9 (4)
N6—Co1—N1—C4	88.5 (3)	N2—C3—N11—C2	−0.7 (4)
N2—Co1—N1—C4	0.4 (2)	C4—C3—N11—C2	176.9 (4)
N3—Co1—N2—C3	92.5 (3)	C3—N11—C2—C1	0.7 (5)
N6—Co1—N2—C3	−93.8 (3)	N11—C2—C1—N2	−0.4 (5)
N1—Co1—N2—C3	1.0 (2)	C3—N2—C1—C2	0.0 (5)
N5—Co1—N2—C3	−175.2 (2)	Co1—N2—C1—C2	−173.3 (3)
N3—Co1—N2—C1	−94.5 (4)	C16—N6—C18—C17	−1.3 (5)
N6—Co1—N2—C1	79.2 (4)	Co1—N6—C18—C17	−169.1 (3)
N1—Co1—N2—C1	174.0 (4)	N6—C18—C17—N7	0.8 (5)
N5—Co1—N2—C1	−2.2 (4)	C18—C17—N7—C16	0.1 (5)
N4—Co1—N3—C9	−0.3 (3)	C17—N7—C16—N6	−0.9 (4)
N1—Co1—N3—C9	−88.9 (3)	C17—N7—C16—C15	174.7 (4)
N5—Co1—N3—C9	89.8 (3)	C18—N6—C16—N7	1.3 (4)
N2—Co1—N3—C9	−171.2 (3)	Co1—N6—C16—N7	172.5 (2)
N4—Co1—N3—C7	−176.5 (4)	C18—N6—C16—C15	−175.2 (3)
N1—Co1—N3—C7	94.9 (4)	Co1—N6—C16—C15	−4.0 (4)
N5—Co1—N3—C7	−86.5 (4)	C13—N5—C15—N8	0.8 (4)
N2—Co1—N3—C7	12.6 (4)	Co1—N5—C15—N8	169.0 (2)
N3—Co1—N4—C10	−3.9 (3)	C13—N5—C15—C16	−177.1 (3)
N6—Co1—N4—C10	−177.1 (3)	Co1—N5—C15—C16	−8.9 (4)
N1—Co1—N4—C10	88.3 (3)	N7—C16—C15—N8	15.6 (7)
N5—Co1—N4—C10	−95.5 (3)	N6—C16—C15—N8	−169.0 (4)
N3—Co1—N4—C12	−167.7 (4)	N7—C16—C15—N5	−167.0 (4)
N6—Co1—N4—C12	19.0 (4)	N6—C16—C15—N5	8.5 (5)
N1—Co1—N4—C12	−75.5 (4)	N5—C15—N8—C14	−0.6 (4)
N5—Co1—N4—C12	100.7 (4)	C16—C15—N8—C14	176.8 (4)
N4—Co1—N5—C15	−90.1 (3)	C15—N8—C14—C13	0.1 (5)
N3—Co1—N5—C15	−172.6 (3)	C15—N5—C13—C14	−0.7 (4)
N6—Co1—N5—C15	5.5 (2)	Co1—N5—C13—C14	−164.5 (3)
N2—Co1—N5—C15	92.8 (3)	N8—C14—C13—N5	0.4 (5)
N4—Co1—N5—C13	72.7 (4)	C10—N4—C12—C11	1.1 (4)
N3—Co1—N5—C13	−9.9 (4)	Co1—N4—C12—C11	165.8 (3)
N6—Co1—N5—C13	168.2 (4)	N4—C12—C11—N9	−1.3 (5)
N2—Co1—N5—C13	−104.4 (4)	C12—C11—N9—C10	1.0 (4)
N4—Co1—N6—C16	88.8 (3)	C11—N9—C10—N4	−0.3 (4)
N1—Co1—N6—C16	178.1 (3)	C11—N9—C10—C9	−175.9 (4)
N5—Co1—N6—C16	−0.7 (3)	C12—N4—C10—N9	−0.5 (4)
N2—Co1—N6—C16	−99.9 (3)	Co1—N4—C10—N9	−169.3 (3)
N4—Co1—N6—C18	−104.1 (4)	C12—N4—C10—C9	176.0 (3)
N1—Co1—N6—C18	−14.8 (4)	Co1—N4—C10—C9	7.1 (4)
N5—Co1—N6—C18	166.5 (4)	C7—N3—C9—N10	0.6 (4)
N2—Co1—N6—C18	67.3 (4)	Co1—N3—C9—N10	−176.9 (2)
C4—N1—C6—C5	−0.4 (4)	C7—N3—C9—C10	−178.5 (3)
Co1—N1—C6—C5	−175.3 (3)	Co1—N3—C9—C10	4.0 (4)
N12—C5—C6—N1	0.5 (4)	N9—C10—C9—N10	−10.7 (8)
C6—C5—N12—C4	−0.3 (4)	N4—C10—C9—N10	173.8 (4)
C5—N12—C4—N1	0.1 (4)	N9—C10—C9—N3	168.2 (4)
C5—N12—C4—C3	177.6 (4)	N4—C10—C9—N3	−7.3 (5)
C6—N1—C4—N12	0.2 (4)	N3—C9—N10—C8	−0.8 (4)
Co1—N1—C4—N12	176.4 (2)	C10—C9—N10—C8	178.1 (4)
C6—N1—C4—C3	−177.9 (3)	C9—N10—C8—C7	0.7 (4)
Co1—N1—C4—C3	−1.7 (4)	C9—N3—C7—C8	−0.1 (4)
C1—N2—C3—N11	0.4 (4)	Co1—N3—C7—C8	176.4 (3)
Co1—N2—C3—N11	175.8 (2)	N10—C8—C7—N3	−0.4 (5)

### Hydrogen-bond geometry (Å, °)

**Table:**

*D*—H···*A*	*D*—H	H···*A*	*D*···*A*	*D*—H···*A*
N11—H6···N12^i^	0.86	1.95	2.808 (4)	172
N7—H11···N8^ii^	0.86	1.99	2.814 (4)	159
N9—H19···N10^iii^	0.86	1.95	2.796 (4)	169

Symmetry codes: (i) −*x*, −*y*+2, −*z*+1; (ii) −*x*, −*y*+1, −*z*+2; (iii) −*x*+1, −*y*+2, −*z*+2.
